# Increasing Quinolone Resistance in *Salmonella* *enterica* serotype Enteritidis

**DOI:** 10.3201/eid0805.010288

**Published:** 2002-05

**Authors:** Kåre Mølbak, Peter Gerner-Smidt, Henrik C. Wegener

**Affiliations:** * Statens Serum Institut, Copenhagen, Denmark; † Danish Zoonosis Centre, Copenhagen, Denmark

**Keywords:** *Salmonella*, *Salmonella* Enteritidis, antimicrobial drug resistance, nalidixic acid, quinolones

## Abstract

Until recently, *Salmonella enterica* serotype Enteritidis has remained sensitive to most antibiotics. However, national surveillance data from Denmark show that quinolone resistance in *S.* Enteritidis has increased from 0.8% in 1995 to 8.5% in 2000. These data support concerns that the current use of quinolone in food animals leads to increasing resistance in *S*. Enteritidis and that action should be taken to limit such use.

*Salmonella enterica* serotype Enteritidis is the most common cause of foodborne salmonellosis worldwide. Historically, this serotype has remained sensitive to most antibiotics, unlike other common serotypes such as Typhimurium, Hadar, Virchow, and Infantis, in which resistance to a wide range of antimicrobial agents is common (*1*)*.* Recently in Denmark, we have recorded increasing resistance to quinolones in *S.* Enteritidis from human infections. This finding is cause for concern because fluoroquinolones are first-line drugs for treatment of human salmonellosis.

## <H1>The Study

From 1995 to 2000, 13,334 *S*. Enteritidis infections were recorded in Denmark, accounting for 62% of all zoonotic salmonella infections. To monitor drug resistance (*2*), we examined a random sample of 2,546 isolates, of which 82 (3.2%) were resistant to the quinolone nalidixic acid. These data showed that quinolone resistance increased from 0.8% (3 of 384 isolates) in 1995 to 8.5% (31 of 366) in 2000 (Figure). Resistance to other antimicrobial agents was infrequent, and quinolone resistance was mainly present as a single resistance.

**Figure:**
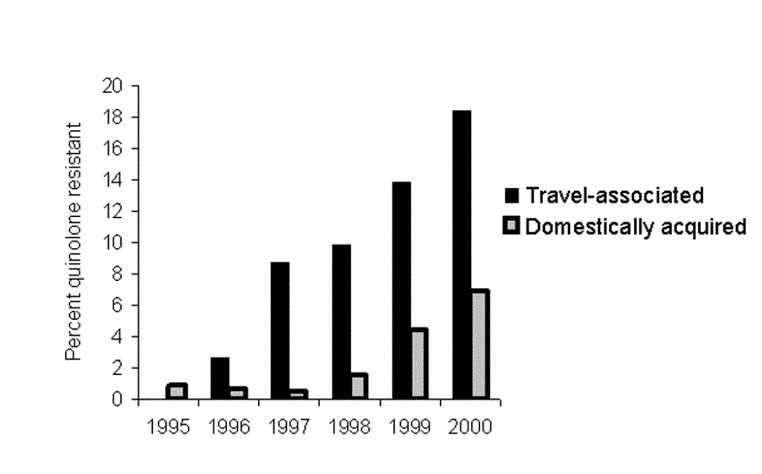
Annual proportion of quinolone resistance in isolates of *Salmonella* Enteritidis, Denmark, 1995–2000.

Quinolone resistance was related to foreign travel as well as *S.* Enteritidis phage type (PT). In isolates from patients with a known history of foreign travel, 8.9% were resistant, compared with 2.4% in domestically acquired infections (p<0.0001; Table). In 157 patients who had returned from a European destination (excluding Scandinavia), 18 (11.5%) had resistant isolates. Resistance was highest in patients returning from Spain: 12 (19.7%) of 61 isolates were resistant. Five (7.5%) resistant strains were found in 67 isolates from Asia (mainly Turkey and Thailand), but no resistant strains were recovered from 25 persons who had traveled to Africa. Five patients had visited other countries (1 resistant strain), and we had no information about the destination for the remaining 48 patients (3 resistant strains).

The major sources of domestically acquired *S.* Enteritidis infections are raw or undercooked eggs produced in Denmark (*3*, unpub. data). The most common phage types in Danish layer hens are PT 6 and PT 8, which accounted for 65.1% of the domestically acquired infections in our study. Resistance in these two phage types remained low (Table), as were the rates of resistance in PT 13A, PT 25, and PT 34. These types also originate from layer hens. In contrast, the proportion of resistant isolates was highest in phage types PT 1, PT 4, PT 6A, PT 14B, and PT 21, which are often associated with infections from imported poultry products, including imported broiler chickens.

**Table:** Prevalence of quinolone resistance in human isolates of *Salmonella enterica* serotype Enteritidis, by phage type and history of foreign travel. Denmark, 1995–2000.

Phage type	No history of foreign travel		Foreign travel	
	No. resistant (%)	Total	No. resistant (%)	Total
1	22 (23.4)	94	11 (29.7)	37
4	7 (2.4)	292	5 (3.8)	131
6	4 (0.5)	765	0 (-)	28
6A	1 (5.9)	17	4 (22.2)	18
8	8 (1.3)	627	0	31
13A	0 (-)	9	0	4
14B	1 (20.0)	5	1 (14.3)	7
21	3 (4.9)	61	0	4
21B	0 (-)	16	0	0
25	0 (-)	19	0	0
34	0 (-)	81	0	2
Others and nontypeable	6 (3.9)	152	4 (13.8)	29
Not typed	2 (1.9)	105	2 (18.2)	11
Total	54 (2.4)	2,243	27 (8.9)	302

From 1994 to 1997 in England and Wales, quinolone resistance in *S.* Enteritidis increased from 0.4 % to 1.3%. As in our study, resistance was highest in PT 1 (19%) and PT 6A (14%) (*4*). These types were mainly associated with foreign travel. In a recent study from Spain, 31% of 385 *S.* Enteritidis isolates overall but 80% of PT 1 isolates were reported to be quinolone resistant (*5*).

## <H1>Conclusions

The emergence of quinolone resistance in the most common salmonella serotype worldwide is a serious public health concern. Resistance to nalidixic acid has been associated with reduced efficacy of fluoroquinolones such as ciprofloxacin (*6*,*7*). The use of nalidixic acid or fluoroquinolones in humans is unlikely to contribute substantially to the increase in resistance, for the following reasons: An antibiotic prescribed in connection with a physician’s request for a fecal specimen is unlikely to have affected the resistance pattern because treatment is usually initiated after the specimen is collected. Fluoroquinolones are potent bactericidal drugs and are not likely to select for resistance when therapeutic concentrations are obtained (*8*). Nalidixic acid is used in some developing countries for the treatment of dysentery, but the this practice is unlikely to select for quinolone-resistant *S.* Enteritidis in the zoonotic reservoir. The prevalence of resistance in *S.* Enteritidis was, in our study, highest in patients returning from developed countries. Furthermore, fluoroquinolones are not used to treat children. In children <15 years of age, the prevalence of quinolone-resistant strains was 9.5% (4/42) among patients with a history of foreign travel and 1.4% (7/499) in domestically acquired cases. The corresponding figures for adults were 8.9% (23/260) and 2.7% (47/1,744). Finally, the use of quinolones in humans could not conceivably be responsible for the large variation in the prevalence of resistance by phage type. If the use of quinolones in human medicine contributed to the emergence of quinolone resistance in *S.* Enteritidis, resistance would be found independently of phage type.

Increasing quinolone resistance is not confined to foodborne salmonella but also includes campylobacters; resistance is primarily driven by the use of fluoroquinolones in the livestock production (*8*,*9*). Limited quantities of fluoroquinolones are currently used in food production in Scandinavia. During 1997-1998, the annual use of the liquid formulation of fluoroquinolones for 130 million to 140 million poultry was <150 kg; during 1999-2000, usage decreased to <100 kg (*2*). Unfortunately, quantitative data on the use of fluoroquinolones are not available from most areas. Several fluoroquinolones are licensed and used in other countries of Europe, Southeast Asia, and the Americas for treatment of food animals, particularly for mass medication in the poultry industry, mainly for broiler chickens (*2*,*7*–*11*). Our data support concerns that the current pattern of quinolone use in food animals leads to increasing quinolone resistance in *S.* Enteritidis and that action should be taken to limit this use.
